# Compulsivity in anorexia nervosa: a transdiagnostic concept

**DOI:** 10.3389/fpsyg.2014.00778

**Published:** 2014-07-17

**Authors:** Lauren R. Godier, Rebecca J. Park

**Affiliations:** Oxford Brain-Body Research into Eating Disorders, Department of Psychiatry, University of OxfordOxford, UK

**Keywords:** anorexia nervosa, compulsivity, obsessive–compulsive disorder, addiction, neurobiology, habit formation, reward

## Abstract

The compulsive nature of weight loss behaviors central to anorexia nervosa (AN), such as relentless self-starvation and over-exercise, has led to the suggestion of parallels between AN and other compulsive disorders such as obsessive–compulsive disorder (OCD) and addictions. There is a huge unmet need for effective treatments in AN, which has high rates of morbidity and the highest mortality rate of any psychiatric disorder, yet a grave paucity of effective treatments. Viewing compulsivity as a transdiagnostic concept, seen in various manifestations across disorders, may help delineate the mechanisms responsible for the persistence of AN, and aid treatment development. We explore models of compulsivity that suggest dysfunction in cortico-striatal circuitry underpins compulsive behavior, and consider evidence of aberrancies in this circuitry across disorders. Excessive habit formation is considered as a mechanism by which initially rewarding weight loss behavior in AN may become compulsive over time, and the complex balance between positive and negative reinforcement in this process is considered. The physiological effects of starvation in promoting compulsivity, positive reinforcement, and habit formation are also discussed. Further research in AN may benefit from a focus on processes potentially underlying the development of compulsivity, such as aberrant reward processing and habit formation. We discuss the implications of a transdiagnostic perspective on compulsivity, and how it may contribute to the development of novel treatments for AN.

Anorexia nervosa (AN) is a severely debilitating psychiatric disorder characterized by relentless self-starvation with dramatic physiological and psychological effects. It is associated with low rates of recovery (Berkman et al., 2007), and has the highest mortality rate of any psychiatric disorder (Arcelus et al., 2011). Individuals with AN place extreme over-importance on the control of weight and shape, which becomes central to their self-evaluation, and often have disturbed body image perception (Fairburn et al., 2003). These distorted beliefs and perceptions are accompanied by a lack of concern over extreme emaciation, a perpetual drive for thinness and continuous lowering of weight goals (Barbarich-Marsteller et al., 2011; see **Figure 1** for DSM-V diagnostic criteria). Characteristic behaviors seen in AN to achieve these goals, such as extreme dietary restriction and driven over-exercise, have been described as evidence of compulsivity and aberrant reward processing (Park et al., 2011, 2012; Cowdrey et al., 2013; Kaye et al., 2013a). Indeed the stereotyped and often ritualistic behaviors seen in AN have been compared to that of obsessive–compulsive disorder (OCD; Steinglass and Walsh, 2006), with the two disorders often being reported as comorbid (Halmi et al., 1991), leading to the suggestion that they may share common underlying neurobiological mechanisms (Steinglass and Walsh, 2006). Simultaneously, parallels with addictive disorders such as substance dependence have been increasingly suggested (Zink and Weinberger, 2010; Barbarich-Marsteller et al., 2011; Kaye et al., 2013a), with similarities in the inability to cease behaviors despite adverse consequences.

**FIGURE 1:**
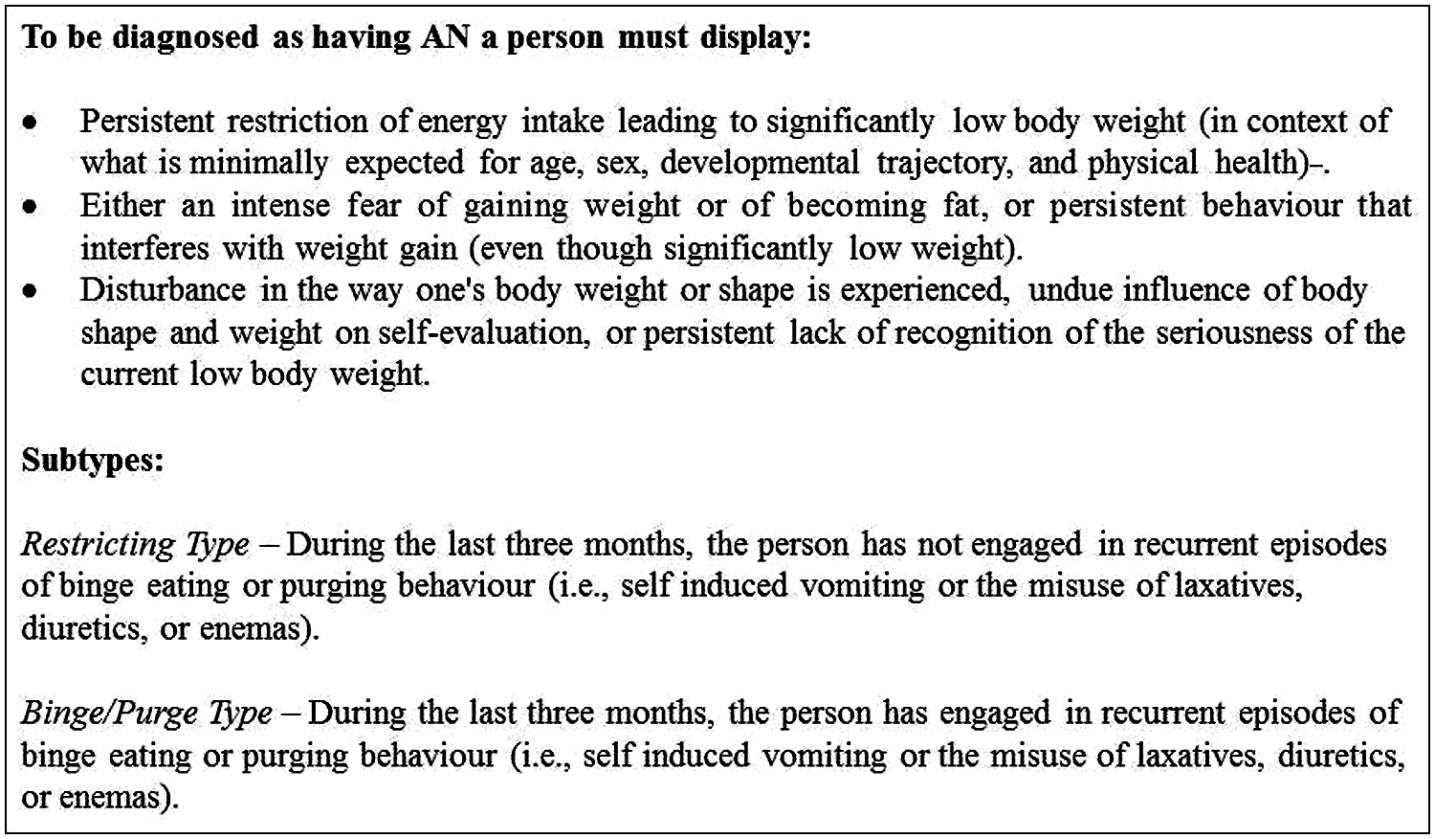
**The DSM-V diagnostic criteria for anorexia nervosa (American Psychiatric Association, 2013)**.

The aim of this review is to explore how a transdiagnostic view of compulsivity, as a dimension on which several psychiatric disorders may fall, can further our understanding of persistent weight loss behavior in AN. Neurobiological and behavioral correlates of compulsivity will be discussed, with particular focus on how these relate to AN, and parallels with OCD and substance dependence. Better understanding of neurobiological and behavioral processes underpinning compulsive weight loss behavior may aid development of much needed novel treatment strategies for AN.

## COMPULSIVE BEHAVIOR IN AN

Compulsivity can be defined as a trait leading to behavior that is inappropriate to the situation, persists despite having no relationship with any overall goal, and results in undesirable consequences (Dalley et al., 2011). In individuals with AN, dietary restriction tends to take on a driven and compulsive quality. This behavior may be motivated by an aberrant sense of reward, specifically the perceived reward of extreme dietary control and thinness (Fladung et al., 2010, 2013; Park et al., 2011, 2012). In some individuals, such extreme control of eating and weight cannot be sustained, and AN may then be complicated by the development of binge eating and compensatory purging such as self-induced vomiting and/or laxative abuse (Fairburn et al., 2003), which also appears to have an element of compulsivity. Compulsive over-exercising is also a common feature, and is reported to be more prevalent in restrictive AN (80%), compared to the binge/purging subtype (43%; Dalle Grave et al., 2008). The presence of compulsive exercise in AN is extremely challenging to manage and can contribute to medically dangerous degrees of weight loss.

The compulsive behaviors seen in AN have often been compared to those of OCD, but with the obsessional focus being on eating, weight and shape. The compulsions that characterize OCD are defined as repetitive, purposeful actions, which are often performed to reduce the anxiety caused by persistent, intrusive thoughts (Steinglass and Walsh, 2006). In the same way, individuals with AN have persistent, intrusive thoughts regarding food and weight gain, and may develop compulsive, ritualized behaviors in an attempt to neutralize the anxiety associated with these thoughts (Steinglass and Walsh, 2006).

Comorbidity is found to be high between AN and OCD (Halmi et al., 1991) OCD is reported to be most prevalent in the restrictive subtype of AN (Fornari et al., 1992; Lilenfeld et al., 1998), although reported prevalence has been inconsistent across studies (Godart et al., 2002). The presence of obsessive–compulsive symptoms is a risk factor for developing AN (Anderluh et al., 2008); and the level of such symptoms remains elevated to some extent even after recovery (Holtkamp et al., 2005). Familiality is reported, with the first degree relatives of individuals with AN showing an elevated risk for OCD (Bellodi et al., 2001). Candidate gene studies suggest common genetic liability between the two disorders (Mas et al., 2013). Comorbidity with obsessive–compulsive personality disorder (OCPD) is also high (Lilenfeld et al., 2006), and the excessive self-control (Pinto et al., 2014), perfectionism and rigidity seen in OCPD (Ansell et al., 2010) may parallel AN more closely.

Aspects of the compulsive behaviors characteristic of AN have increasingly been compared to the compulsive drug-seeking behavior seen in substance dependence (Scheurink et al., 2010; Zink and Weinberger, 2010; Barbarich-Marsteller et al., 2011; Kaye et al., 2013a). The developmental period of onset is similar, with an initial phase of reward seeking, in the form of weight loss in AN, which is experienced as rewarding and pleasurable (Scheurink et al., 2010; Park et al., 2011, 2012), as if it were a drug. This is followed by a narrowing of the behavioral repertoire and the lack of ability to cease behaviors despite their adverse consequences (Kalivas and Volkow, 2005). The compulsive drug-seeking behavior of addicts may parallel the relentlessness with which individuals with AN pursue weight loss. Individuals find it increasingly difficult to refrain from weight loss behavior such as restriction and compulsive exercise despite adverse consequences, and even describe symptoms of withdrawal similar to those experienced in drug addiction (Allegre et al., 2006). In terms of comorbidity, there is a higher incidence of substance dependence in ED than the general population: for example, the US National Centre on Addiction and Substance Abuse found that up to 50% of individuals with an eating disorder abuse substances compared with 9% of the general population, and up to 35% of individuals with substance abuse have an eating disorder compared with 3% of the general population (Casa, 2003; Baker et al., 2013). However, a lower incidence of substance use has been reported in restrictive AN than in other types of eating disorders such as bulimia nervosa (BN) (Holderness et al., 1994; Kaye et al., 2013a). This suggests that there may be a specific relationship between the binge-purge cycle of behavior, which may itself take on an addictive quality, and higher rates of substance abuse (O’Brien and Vincent, 2003).

Nevertheless, there are notable distinctions in information processing between AN, OCD, and substance dependence. In AN, an increased focus on delayed gratification and long term goals is seen (Kaye et al., 2013a). This is reflected in marked differences in the ability to delay reward in AN as compared to substance dependence. Substance dependent individuals and those with binge eating disorder show a preference for smaller immediate reward (Davis et al., 2010), whereas individuals with AN favor delayed larger reward (Steinglass et al., 2012b). That said, starvation in those vulnerable to AN may produce an immediately rewarding sense of control (Park et al., 2012), acting as a positive reinforcer of behavior. Equally, avoiding negative consequences such as dysphoric mood during refeeding, which some individuals with AN experience as “withdrawal symptoms” from starvation, may be important short term goals. Perhaps as a consequence of these immediate reinforcers, the long term goal of weight loss becomes irrationally overvalued (Barbarich-Marsteller et al., 2011). Interestingly, individuals with OCPD are more able to delay reward than those with OCD, and this ability to delay reward is associated with perfectionism and rigidity (Pinto et al., 2014). This supports the suggestion that AN may parallel OPCD more closely than OCD. That said, studies looking at decision making processes in AN, OCD, and substance dependence suggest in all three disorders a tendency to make disadvantageous decisions when choosing between immediate or long terms gains (Lawrence et al., 2006; Tchanturia et al., 2007b; Verdejo-Garcia et al., 2007). This impairment in decision making is suggested to be linked to the compulsive and self-destructive behavior seen across these disorders (Tchanturia et al., 2007b). Whilst individuals with AN may show the ability to delay reward in general, their impairment in decision making may lead them to engage in compulsive weight loss behaviors despite adverse outcomes.

### COMPULSIVITY AS A TRANSDIAGNOSTIC CONCEPT

The core feature that unites AN, OCD, and substance dependence is the compulsive nature of disorder-specific behavior. Compulsive weight loss behavior, such as persistent food restriction and over-exercise, is a prominent feature of AN, and is parallel to the compulsive behaviors characteristic of OCD and substance dependence; with inevitable comorbidities. If such parallels reflect similarities in the underlying mechanisms that drive this behavior, there should be some agreement about the neurobiological correlates of aberrant behavior across these disorders. Robbins et al. (2012) have suggested a transdiagnostic approach to compulsivity, arguing that it has cross-diagnostic significance, as evidenced by commonalities and comorbidities in behaviors across a range of disorders. They posit that a transdiagnostic approach to compulsivity may aid in the development of novel treatment avenues relating to specific behaviors, rather than focusing on diagnosis. This focus on constructs of behavior, as opposed to symptoms and disorder categories, reflects the recent RDoC (Research Domain Criteria) research strategy adopted by the National Institute of Mental Health (NIMH). This strategy emphasizes the need to break away from the use of symptoms and diagnostic categories for classification. Instead, research should focus on the variables that define certain dimensions of behavior, or constructs, seen transdiagnostically across psychiatric disorders. In line with this, we suggest that focusing on compulsivity as a transdiagnostic concept may help in understanding commonalities in the compulsive behaviors seen not only in AN but also in other disorders, such as OCD and substance dependence, without categorizing them together unnecessarily.

There is a huge unmet need for translational research in AN to develop novel treatments, especially for adults with severe AN. Effective treatments require an optimal understanding of processes underlying AN, so that the correct treatment targets are identified. The use of a transdiagnostic approach to compulsive weight loss behaviors in AN may begin to address this problem. This review will now focus on the neurobiological and behavioral correlates of compulsivity across disorders and how this can guide novel avenues for the development of treatments for AN.

### SUMMARY

• Parallels are seen between the compulsive nature of behavior in AN, OCD, and substance dependence.
• Despite similarities, important distinctions in disorder-specific compulsivity and information processing are seen across disorders.
• A transdiagnostic view of compulsivity, seen in varying manifestations across disorders, may aid the development of treatments targeting compulsive behavior.

## WHAT IS THE NEURAL BASIS OF COMPULSIVITY?

**FIGURE 2:**
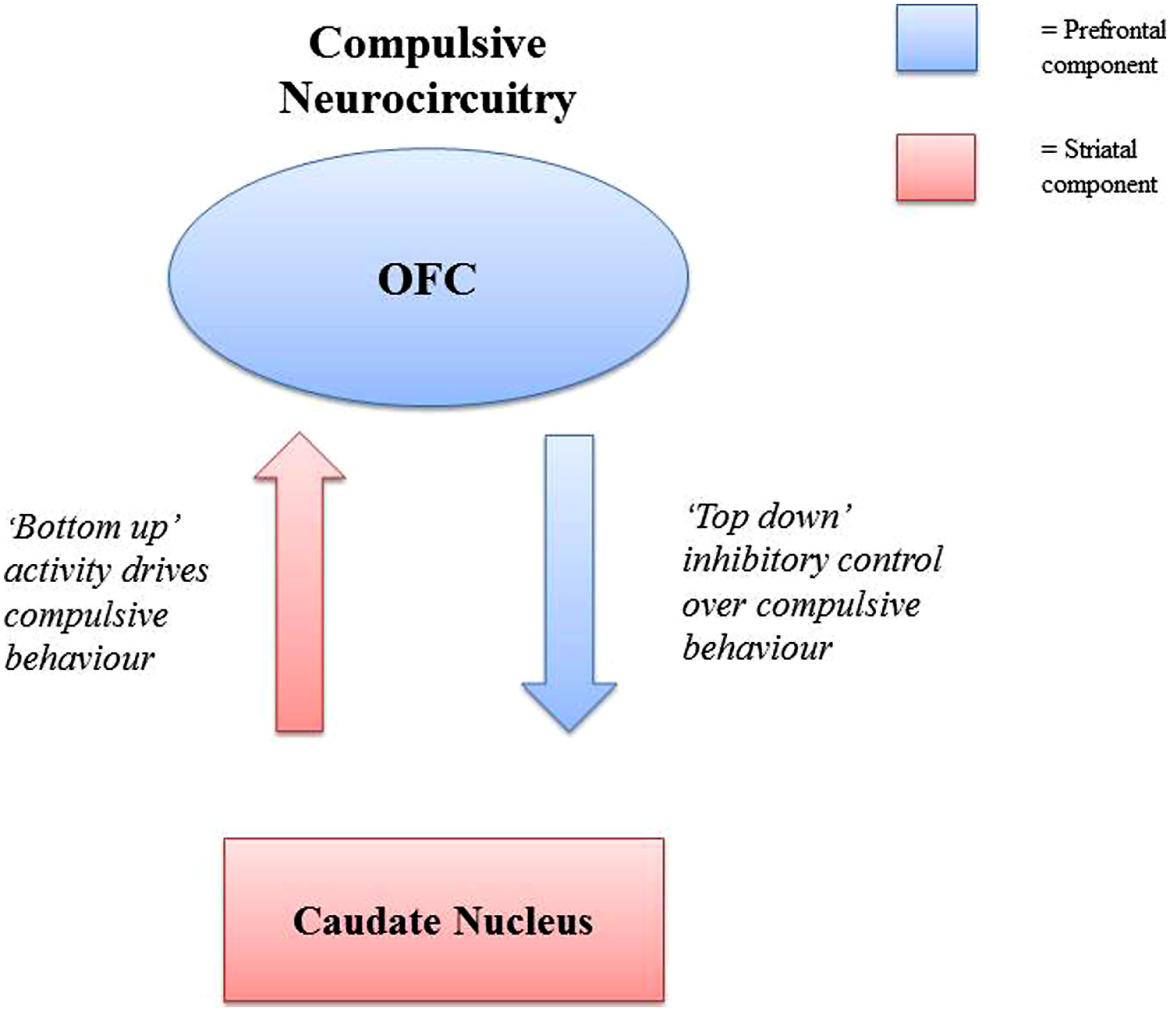
**Representation of the cortico-striatal circuitry suggested to be involved in the development compulsive behavior (Robbins, 2007; Brewer and Potenza, 2008; Fineberg et al., 2010).** In the circuit, activity in the striatal component drives compulsive behavior, whilst activity in the prefrontal component inhibits compulsive behavior. Both failures in “top down” control of the prefrontal components, and over activity of “bottom up” striatal activity can result in increases in compulsive behavior. Abbreviation: OFC, orbitofrontal cortex.

Models of the neurocircuitry involved in compulsive behavior suggest the involvement of a cortico-striatal circuit, consisting of a striatal and prefrontal component (Robbins, 2007; Brewer and Potenza, 2008; Fineberg et al., 2010). The striatal component is seen as responsible for driving compulsive behavior, while the prefrontal component controls or inhibits this behavior. Abnormalities in either of these components (hypoactivity/hyperactivity) may result in an increase in compulsive behavior. The caudate nucleus (the striatal component) is suggested to drive compulsive behaviors, with the orbitofrontal cortex (OFC, the prefrontal component) exerting inhibitory control over these (see **Figure 2**). This is distinct but related to an “impulsive” cortico-striatal circuit, involving the ventral striatum (VS)/nucleus accumbens (NAc) (striatal component), and the anterior cingulate cortex (ACC)/ventromedial prefrontal cortex (vmPFC; prefrontal component). These compulsive and impulsive circuits are suggested to be intercommunicating, with the possibility that abnormalities in one circuit lead to abnormalities in the other (Fineberg et al., 2010). This is relevant for theories of dependence in which it is suggested that what may begin as impulsive behavior may eventually become compulsive with repetition of behavior, with a corresponding shift in control from impulsive to compulsive neural circuitry (Everitt and Robbins, 2005). This may relate to eating disorders involving binge/purge behaviors, in which the behaviors may initially be driven by impulsivity rather than compulsivity.

This compulsive cortico-striatal circuit can be illustrated using tasks which are thought to measure compulsivity. Failures in reversal learning, the ability to adapt behavior following negative feedback, have been suggested to reflect cognitive inflexibility, meaning a rigid cognitive style, which may contribute to compulsivity (Fineberg et al., 2010). Reversal learning is impaired by lesions to the OFC (Dias et al., 1996; Berlin et al., 2004; Remijnse et al., 2006), an area selectively activated during reversal learning tasks (Hampshire and Owen, 2006). Lesions to the medial striatum have also been shown to produce impairments in reversal learning in monkeys (Clarke et al., 2008), supporting the suggestion that a cortical and striatal component is involved in compulsive behavior. Set-shifting tasks, in which attention is required to switch between multiple tasks, or elements of a task (Miyake et al., 2000), is also thought to measure cognitive inflexibility (Fineberg et al., 2010). Lesions to the lateral PFC in primates (Dias et al., 1996) and the ventrolateral PFC in humans (Hampshire and Owen, 2006) impair performance on set-shifting tasks. Failures in both these types of task, and the corresponding neuroanatomy, suggest that impairments in “top down” cortical control may underpin compulsive behavior, and over activity of striatal regions may also underpin, or exacerbate compulsivity.

### ARE NEURAL AND BEHAVIORAL CORRELATES OF COMPULSIVITY OBSERVED IN AN, OCD, AND ADDICTION?

Dysfunction of the neurocircuitry implicated in compulsivity can be seen across AN, OCD, and addiction (see **Table 1**). In terms of a dysfunctional striatal component, AN has been associated with increased caudate function, measured both directly during a monetary reward task (Wagner et al., 2007) and indirectly during exposure to aversive food stimuli (Cowdrey et al., 2011). In OCD, increased functional connectivity within the cortico-striatal circuitry has been shown to correlate positively with Y-BOCS score, together with increased volume and activity in the caudate nucleus (Hou et al., 2013). Evidence of increased activity in the caudate nucleus (Saxena et al., 1998) and alterations in caudate volume have also been reported in OCD (Scarone et al., 1992). In contrast to AN and OCD, chronic substance use has been associated with decreased activation of the caudate during reward anticipation (van Hell et al., 2010). However, high scores on self-reported food addiction have been associated with increased activation in the caudate (Gearhardt et al., 2011), suggesting there may be differences in caudate activation between behavioral and substance addictions.

**Table 1:** Evidence of abnormalities in the neurocircuitry, neurotransmitters and behavioral correlates of compulsivity, across AN, OCD, and Addictions.

	Model of compulsivity	Evidence in AN	Evidence in OCD	Evidence in addictions
Neurocircuitry	• Fronto-striatal circuit involving the OFC (prefrontal component) and the caudate nucleus [striatal component; Robbins (2007), Brewer and Potenza (2008), Fineberg et al. (2010)]. • Abnormalities result from impairments in prefrontal inhibitory control OR over activity of the striatal component. Robbins (2007), Brewer and Potenza (2008), Fineberg et al. (2010).	• Increased caudate function during reward task Wagner et al. (2007), and during exposure to aversive food stimuli Cowdrey et al. (2011). • Decreased OFC activity during disorder related stimuli Uher et al. (2004), decreased OFC volume Frank et al. (2013). • Deactivation of fronto-striatal circuitry predicted by levels of compulsivity Rothemund et al. (2011).	• Increased functional connectivity of CTSC circuits correlated with Y-BOCS scores Hou et al. (2013). • Increased volume and activity in caudate Scarone et al. (1992), Saxena et al. (1998), Hou et al. (2013). • Reduced activation of OFC during reversal learning Chamberlain et al. (2008); abnormalities in OFC gray matter Menzies et al. (2008), reduced functional connectivity of OFC Meunier et al. (2012).	• Increased functional connectivity of CTSC circuits correlated with Y-BOCS scores Hou et al. (2013). • Increased volume and activity in caudate Scarone et al. (1992), Saxena et al. (1998), Hou et al. (2013). • Reduced activation of OFC during reversal learning Chamberlain et al. (2008); abnormalities in OFC gray matter Menzies et al. (2008); reduced functional connectivity of OFC Meunier et al. (2012).
Neurotransmitters	• Role for serotonin and 5-HT receptors: impairments in reversal learning linked to reduced serotonin Clarke et al. (2004), Evers et al. (2005); antagonizing 5HT-2A receptors impairs reversal learning Boulougouris et al. (2008). • Role for the mesolimbic dopamine system: DA tone in the ventral loops important in regulating reward and reinforcement Wise (2002), Fineberg et al. (2010); DA D2 receptor agonists associated with impairments in reversal learning Cools et al. (2006), and increased compulsive behavior in Parkinson’s disease Voon et al. (2009). • Modulation of glutamate by DA involved in reward reinforcement processes Guardia et al. (2011), glutamate involvement in set-shifting Nicolle and Baxter (2003).	• Increased 5HT-1A and decreased 5HT-2A binding potential Kaye et al. (2005b), Bailer et al. (2007). • Altered striatal DA release in recovered AN: increased binding of DA D2/3 receptors in anterior VS Frank et al. (2005), Bailer et al. (2013); DA release experienced as anxiogenic Bailer et al. (2012). • DA D2 receptor polymorphism linked to AN Bergen et al. (2005). • Reduced HVA in CSF in recovered AN Kaye et al. (1999), increased levels in acute AN Castro-Fornieles et al. (2008). • Increased CSF glutamate in AN Nakazato et al. (2010).	• Agonizing 5HT-2C and 1B receptors increases compulsivity in OCD Hollander et al. (1991), Gross-Isseroff et al. (2004), Tsaltas et al. (2005). • DA D2 receptor agonists linked to repetitive checking behavior in rodents Pitman (1989), Tizabi et al. (2002). • Successful use of antipsychotics in treating OCD patients Cavedini et al. (2004). • Increased levels of DA in the striatum Perani et al. (2008). • Increased CSF glutamate in OCD patients; efficacy of drugs that act on GABA and glutamate pathways Pittenger et al. (2011).	• Antagonizing 5HT-2A and agonizing 5HT-2C receptors prevents relapse in rodents Fletcher et al. (2002), Pentkowski et al. (2010); antagonizing 5HT-1B receptors inhibits cocaine-seeking in rodents Miszkiel et al. (2011). • Initial reinforcing effect of drugs associated with release of extracellular DA in striatum Koob and Volkow (2010). • Decreased activity in mesolimbic DA system with chronic use Volkow et al. (2007a); Decreased DA D2 receptors and DA release in striatum Volkow et al. (2002). • Efficacy of drugs that act on GABA and glutamate pathways Kenny and Markou (2004).
Behavioral tasks	• Impairments in cognitive flexibility tasks such as reversal learning Fineberg et al. (2010), associated with OFC Dias et al. (1996), Berlin et al. (2004), Remijnse et al. (2006) and medial striatal abnormalities Clarke et al. (2008), and set-shifting Fineberg et al. (2010) associated with PFC abnormalities Dias et al. (1996), Hampshire and Owen (2006).	• Low in cognitive flexibility Tchanturia et al. (2004). • Impairments on set-shifting tasks Holliday et al. (2005), Steinglass et al. (2006), Bühren et al. (2012). • Subtle impairments in reversal learning Sarrar et al. (2011).	• Impairments on set-shifting Fontenelle et al. (2001), Lawrence et al. (2006) and reversal learning tasks Remijnse et al. (2006), Chamberlain et al. (2008), Valerius et al. (2008).	• Impairments on set-shifting Saraswat et al. (2006) and reversal learning tasks Izquierdo and Jentsch (2012).

Decreased activation of a prefrontal component can also be seen across disorders. Reduced activity in the OFC whilst viewing disorder-related stimuli has also been found in AN (Uher et al., 2004), and alterations in OFC volume has been reported (Frank et al., 2013). Reduced activation of the OFC during reversal learning has been found in individuals with OCD and their unaffected relatives (Chamberlain et al., 2008), and abnormalities in OFC gray matter have been found in both OCD (Menzies et al., 2008), and substance dependence (Franklin et al., 2002) Reduced functional connectivity of the right inferior and superior OFC has been related not only to self-reported compulsivity in OCD but also to compulsive drug taking in substance dependence (Meunier et al., 2012).

Abnormalities in the “compulsive” circuit are implied by deficits in neuropsychological tasks thought to tap this construct (see **Table 1**). Individuals with AN are often described as having low cognitive flexibility (Tchanturia et al., 2004), and consistently show poor set-shifting abilities (Holliday et al., 2005; Steinglass et al., 2006; Bühren et al., 2012). Poor set-shifting has been suggested as an endophenotype of both AN and BN, and is associated with both longer illness duration and increased disorder-related rituals (Roberts et al., 2010). Subtle impairments in reversal learning have also been reported in AN, and are shown to occur before and after weight gain (Sarrar et al., 2011), suggesting this is a trait rather than state related impairment. OCD and substance dependence have also both been associated with deficits in reversal learning (Remijnse et al., 2006; Chamberlain et al., 2008; Valerius et al., 2008; Izquierdo and Jentsch, 2012), and set-shifting (Fontenelle et al., 2001; Lawrence et al., 2006; Saraswat et al., 2006).

### NEUROTRANSMITTER INVOLVEMENT IN COMPULSIVITY

#### The role of serotonin

Serotonin has widespread effects on satiety, impulse control and mood, with a range of evidence indicating a specific role for 5-HT receptors in compulsive behavior (see **Table 1**). A rodent model of OCD indicates that 5-HT-2C receptor agonists increase compulsivity or the persistence of response in these rats (Tsaltas et al., 2005). This result has been replicated in exacerbating compulsive symptoms in human OCD patients (Hollander et al., 1991), in whom 5-HT-1B receptor agonists also exacerbate OCD symptoms (Gross-Isseroff et al., 2004). Impairments in reversal learning have been linked to reduced brain serotonin (Evers et al., 2005), particularly in areas such as the OFC (Clarke et al., 2004), and antagonism of 5-HT-2A receptors impairs reversal learning (Boulougouris et al., 2008), indicating their involvement in the development of compulsive behavior.

The 5-HT system has been extensively studied in AN, with much evidence of dysfunction in this system (for a recent review see Kaye et al., 2013b). Imaging studies have consistently shown increased 5HT-1A and decreased 2A receptor binding potential in individuals both currently ill and recovered from AN (Kaye et al., 2005a,b; Bailer et al., 2007), suggesting a trait and not state related alteration. Interaction between 5HT-1A and 2A receptors in the medial prefrontal cortex (mPFC) have been suggested to modulate impulsivity and compulsivity (Carli et al., 2006), although there is yet to be evidence of this mechanism in the development of compulsive behavior in AN.

The 5-HT system is modulated by many drugs of abuse (Kirby et al., 2011). Research in this area is extensive, the scope of which cannot be covered in this review. However, evidence to date points to a particular role for 5HT-2A and 2C receptors in relapse during withdrawal, and in the persistence of compulsive drug-seeking in addiction (Fletcher et al., 2002; Pentkowski et al., 2010). Furthermore, there is an inhibitory effect of 5-HT-1B receptor antagonists on cocaine-seeking behavior (Miszkiel et al., 2011). In terms of behavioral addictions, peripheral measures suggest reduced serotonin levels in pathological gamblers (Nordin and Sjödin, 2006). This has been hypothesized to be linked to maladaptive decision making in these individuals (Kirby et al., 2011), and treatment with SSRIs has been reportedly effective (Hollander and Rosen, 2000). Although aberrancies in the 5-HT system are seen across compulsive disorders, these do not appear to be universal in nature, suggesting that the serotonergic neurotransmitter system alone is not driving compulsive behavior.

#### The role of dopamine

Dopamine (DA) is considered key to the rewarding effects of both natural and drug-derived reward (Volkow et al., 2012b), and the mesolimbic DA pathways in particular play a crucial role in reward and reinforcement processes (Wise, 2002). Models of compulsivity have emphasized the importance of DA tone in the ventral loops that link the ventral ACC and the VS/NAc in regulating reward and reinforcement behaviors (Fineberg et al., 2010; see **Table 1**). DA D2 receptor agonists such as levodopa have been associated with deficiencies in reversal learning (Cools et al., 2006), and increased compulsive behavior in Parkinson’s disease (Voon et al., 2009). Studies in animals have also shown that the administration of DA agonists induces stereotyped behaviors associated with OCD (Pitman, 1989). Specifically, the use of a selective D2 receptor agonist in rats was associated with the development of repetitive checking behavior (Tizabi et al., 2002). DA dysfunction in OCD patients is also indicated by the successful use of antipsychotics when SSRI treatment is unsuccessful (Cavedini et al., 2004). There have also been reports of increased levels of DA in the striatum in OCD (Perani et al., 2008).

A key process in the initial reinforcing effect of drugs is their ability to produce increases in extracellular DA in limbic regions such as the NAc/VS (Koob and Volkow, 2010). This release of DA is associated with a feeling of euphoria, and is experienced as rewarding (Volkow et al., 1996), thus positively reinforcing their use. The expectation of drug reward (i.e., in certain contexts) is also important in reinforcing drug use (Volkow et al., 2003), and is likely to depend on neurotransmitters such as glutamate, which is known to modulate DA release in the NAc (Kalivas and Volkow, 2005). These DA- dependent effects cause drugs themselves and drug-associated stimuli to gain incentive salience and promote further drug-seeking behavior (Everitt and Robbins, 2005). This repeated and prolonged DA increase results in synaptic changes in DA pathways, and these changes may be responsible for the formation of compulsive habits that persist despite adverse consequences in substance dependence (Wolf, 2002).

In substance dependent individuals, activity in the mesolimbic DA system is decreased, a deficiency that persists for months following detoxification (Volkow et al., 2007a). Chronic cocaine abusers are shown to have decreased levels of D2 receptors and a decrease of DA release in the striatum (Volkow et al., 2002), although initial drug use is associated with synaptic increases in DA (Volkow et al., 2007a). Evidence of decreased DA activity following chronic drug use has led to the suggestion that this deficiency in DA may cause an increase in the compulsion to seek further drug reward to order to increase deficient DA levels, with repetition of this behavior leading to a dependence on the substance (Fineberg et al., 2010). In contrast, individuals recovered from AN have increased binding of DA D2/D3 receptors in the anterior VS while currently ill AN patients has shown increased levels of the DA metabolite homovanillic acid (HVA) in cerebrospinal fluid (Castro-Fornieles et al., 2008). The increased DA receptor binding in recovered AN patients contrasts with the reduced striatal DA binding found in those with substance abuse (Volkow et al., 2009, 2012a), which is paralleled by those with BN (Broft et al., 2012), and obesity (Kenny, 2011). Striatal DA response has been negatively associated with binge eating and vomiting (Broft et al., 2012), suggesting that individuals who engage in overeating may have reduced DA function, similar to that found in substance dependence. DA release in the dorsal putamen has been associated with anxiety in recovered AN, which contrasts with the euphoria reported by controls (Bailer et al., 2012). Palatable food ingestion is associated with DA release in the striatum (Bassareo and Di Chiara, 1999), suggesting that individuals with AN may experience this DA release as aversive, as opposed to the hedonic response seen in healthy controls. This aversive reaction to food may explain the relentless food restriction seen in AN (Kaye et al., 2013a).

Glutamate is the principal excitatory neurotransmitter in the brain and is involved in many cognitive functions such as memory and learning (Jamain et al., 2002). The mesolimbic DA system, central to brain reward processes and compulsivity, has a variety of excitatory glutaminergic and inhibitory gamma-amino butyric acid (GABA, of which glutamate is the precursor) inputs (Guardia et al., 2011). The NAc, a region heavily implicated in reward processes, has glutaminergic inputs from limbic regions, and GABAergic projections to other reward-related areas (Guardia et al., 2011). There is extensive physiological and neurochemical evidence to suggest that reward-related learning requires an interaction between DA and glutamate, and occurs as the result of modulation by DA of glutamate synapses in the striatum (Beninger and Gerdjikov, 2005). Alterations in glutamate receptor binding in the cingulate cortex and dorsal striatum has been associated with impairments in set-shifting ability (Nicolle and Baxter, 2003), further suggesting a role for glutamate in cognitive flexibility and compulsivity.

Dopamine modulation of glutamate has been implicated in the development and expression of addictive behaviors, and there is evidence suggesting that modulation of GABA and glutamate pathways may be effective in the treatment of substance use disorders (Olive et al., 2012). Animal studies have shown the efficacy of GABA agonists in attenuating the positive reinforcing effects of drugs, and glutamate antagonists may be effective in preventing relapse (Kenny and Markou, 2004). Drugs modulating glutaminergic and GABAergic pathways have also been shown to act on binge eating, purging and weight loss in eating disorders (Guardia et al., 2011). Interestingly, both OCD and AN are associated with an increase in cerebrospinal fluid glutamate (Nakazato et al., 2010; Pittenger et al., 2011). Although successful treatment with glutaminergic drugs has yet to be reported in AN, some improvement has been found in the treatment of OCD (Pittenger et al., 2011).

### SUMMARY

• Models of compulsivity indicate a bottom up striatal component driving compulsivity, and a top down prefrontal component inhibiting compulsive behavior.
• Dysfunction of this neurocircuitry, and impairments in tasks reflecting activity in these regions, can be seen in AN, OCD, and substance dependence.
• Abnormalities in neurotransmitter activity linked to impulse control and reward, such as DA, glutamate and serotonin, are also seen across disorders.

## DOES COMPULSIVITY REFLECT DYSREGULATED HABIT FORMATION?

Compulsivity, as previously defined, can also be described as a tendency to carry out repetitive acts in a habitual manner (Fineberg et al., 2010). Habits are described as behaviors that are not innate, are engaged in repeatedly and become fixed, occur without conscious effort and can be elicited by external stimuli (Graybiel, 2008). Two distinct types of learning are involved in the development of behavior that is not innate or is outside of conscious awareness: action-outcome learning and stimulus-response learning (Robbins et al., 2012). Action-outcome learning (also referred to as goal-directed learning) occurs when a particular action leads to a rewarding outcome. If at any point the action no longer leads to reward, the frequency of that action will decrease (Balleine and O’Doherty, 2010). However, if these new actions are engaged in repeatedly (over-trained), they may become insensitive to the outcome, and will be repeated even when they do not result in reward (stimulus-response learning; Graybiel, 2008). Thus, behavior can become a habitual response to environmental stimuli associated with the rewarding outcome (Steinglass and Walsh, 2006). Despite the suggested distinction between goal-directed and habit learning, early description of habits describe them as a form of automatic goal-directed behavior. Bargh (1989) suggests that habits form as the instrumental link between goals and actions, and are automatically activated when a relevant goal is present. This may be particularly relevant for disorders such as AN, in which automatic habits, which take the form of compulsive weight loss behaviors, may occur unconsciously in the persistent presence of long term weight loss goals.

Research indicates neural distinctions between goal-directed and habit learning. In humans, the ventromedial PFC has been linked to goal directed learning (Daw et al., 2005), while the putamen has been linked to habit learning (Tricomi et al., 2009). Cortico-striatal connectivity as indexed by diffusion tensor imaging (DTI), which measures the strength of white matter tracts has been associated with differences in habit and goal-directed control of actions (de Wit et al., 2012). In a behavioral learning task, the tendency to rely on habits was associated with white matter tract strength between both premotor cortex and posterior putamen, and gray matter density in the posterior putamen; while the tendency to use goal directed control was associated with tract strength in the ventromedial PFC from the caudate (de Wit et al., 2012). The cortico-striatal circuitry implicated in habit vs. goal directed behavioral control is similar to that suggested in models of the neurocircuitry of compulsivity (see **Figure 2**), supporting the suggestion that compulsive behavior may be underpinned by habitual control of behavior, as these constructs appear to be indexed by overlapping neurocircuitry. Investigating abnormalities in habit learning and related brain areas may thus further understanding of the development of compulsive behavior across disorders.

### IS EXCESSIVE HABIT FORMATION OBSERVED IN AN, OCD, AND SUBSTANCE DEPENDENCE?

Emergent evidence suggests that dysregulation of habit formation may provide a mechanism by which the development of well-entrenched, compulsive behaviors can occur: for example, once drug taking is engaged in repeatedly, it becomes associated with a number of environmental stimuli or cues, which thereby become triggers for compulsive drug-seeking and drug taking, despite knowledge of the negative consequences of engaging in this behavior (Belin et al., 2011). Habit learning may also play a role in disorders such as AN and OCD, in which certain compulsive, and sometimes ritualistic, behaviors may persist despite a lack of reward or intermittent reward, and a wide variety of negative consequences (Steinglass and Walsh, 2006).

The development of substance dependence has been described in terms of a transition from initially goal-directed drug taking, driven by the reinforcing properties of the drug, to progressively more compulsive drug-seeking controlled by the habit system (Everitt and Robbins, 2005), and ultimately driven by environmental stimuli associated with the drug (Belin et al., 2011). Animal models of compulsive drug-seeking have investigated habit formation using outcome devaluation paradigms, in which previously rewarding action-outcome contingences are devalued, and persistence of behavior despite this devaluation is measured. These demonstrate that reinforcement with cocaine, in comparison to a natural reward such as lemon-sucrose solution, resulted in accelerated habit learning, and was subsequently less sensitive to devaluation (Miles et al., 2003). Similar results have been found with other drugs of abuse, such as alcohol (Dickinson et al., 2002), suggesting that the reinforcing effects of drugs may promote dominance of the habit system in learning and result in the development of compulsive drug-seeking behaviors. Everitt and Robbins (2005) support this idea with a neural systems model of substance dependence, in which this transition from goal directed to habitual control represents a change from prefrontal cortical to striatal control, and a progression from ventral to dorsal areas of the striatum (Everitt and Robbins, 2005). This is supported by rodent studies (Ito et al., 2000, 2002), and data from human cocaine addicts showing increased dorsal striatum activity during presentation of drug cues (Volkow et al., 2006). Drugs of abuse may also decrease the ability of the individual to exert control over these habits, even when presented with persistent negative and aversive consequences (Deroche-Gamonet et al., 2004). A parallel can be drawn here with the compulsive behaviors seen in AN: individuals with AN often report wanting to recover from their disorder, despite persisting with compulsive behavior that maintains their emaciation.

Compulsive behavior in OCD may also be associated with over-reliance on habits in learning; Gillan et al. (2011) showed that individuals with OCD had a selective impairment in goal directed control of behavior which forced them to rely on previously learned habits. Although individuals with OCD showed no overall impairment in using feedback to guide learning, they showed weaker knowledge of the direct causal relationship between actions and outcomes, leading to errors. Symptom severity was predictive of persistent responding to devalued stimuli, suggesting the continued use of previously learned habits may be related to the severity of compulsions (Gillan et al., 2011). Using a shock avoidance paradigm, Gillan et al. (2013) also demonstrated that individuals with OCD have a tendency to develop excessive avoidance habits, further supporting the idea that a reliance on habit formation in learning at the expense of goal directed control may underlie compulsive behaviors in OCD.

Walsh (2013) elegantly outlines the mechanisms by which aberrant habit formation may contribute to the maintenance of AN. It is suggested that restrictive eating may begin as the result of goal-directed weight loss behavior, in which behavior becomes associated with a rewarding outcome (weight loss). If this restrictive eating behavior is repeated enough it may become relatively insensitive to reward. In this way, weight loss behavior becomes highly practiced and over trained, and weight loss as a rewarding outcome may be needed only intermittently, or even no longer necessary for this behavior to continue. The fact that habitual behavior is outcome-independent makes it highly resistant to change, reflecting the treatment resistance often seen in individuals with AN (Walsh, 2013). It is further suggested that during the process of habit formation in AN, restrictive behavior itself becomes rewarding through conditioned reinforcement (Walsh, 2013), in which a set of cues begin to develop, and take on rewarding properties themselves (Everitt and Robbins, 2005). Individuals with AN may thus start to find that the reward of weight loss is no longer required as the now habitual weight loss behaviors themselves and associated cues have become rewarding or reinforcing. This process is also seen in substance dependence, in which cues associated with drug taking become associated with craving for the drugs and drug taking (Wikler, 1973).

Rothemund et al. (2011) reported that high levels of compulsivity in severely low weight AN patients predicted deactivation of the fronto-striatal circuitry, a finding that was interpreted as reflecting the transition from goal-directed actions to the development of habitual compulsive behavior, similar to that described in substance dependence (Everitt and Robbins, 2005). During the high calorie picture conditions in this study, individuals with AN showed increased activation in the right caudate and right precuneus, suggested to reflect differences in automatic habit learning processes compared to control participants. Furthermore, reduced gray matter volume in the bilateral OFC and right middle and superior frontal gyrus was found in individuals with AN, indicative of dysfunctional goal-directed behavioral control. The results of this study suggest aberrancies in the neurocircuitry associated with goal directed actions and habits in AN, which may reflect the persistence of compulsive weight loss behaviors in this disorder.

### SUMMARY

• Excessive reliance on stimulus-response habits in learning may underpin the development of compulsive behaviors, with evidence of this in both OCD and substance dependence.
• Models of addiction suggest a transition over time from initially goal directed to habitual behavior, reflected by a shift in corresponding neurocircuitry.
• Recent theory implicates habit formation in the development of compulsive weight loss behaviors in AN, but to date there is no published evidence testing this theory.

## THE ROLE OF POSITIVE AND NEGATIVE REINFORCEMENT IN COMPULSIVITY

Whilst AN and OCD have often been conceptualized as disorders driven by avoidance and fear of weight gain in AN, and of disorder related obsessions in OCD- substance dependence is commonly thought to be driven by an approach response, to gain the rewarding effects of drugs. If compulsive behavior is driven by a common process in these disorders, there should be some similarities in the reinforcement processes maintaining compulsive behavior.

### POSITIVE AND NEGATIVE REINFORCEMENT IN AN

AN has often been associated with anhedonia and a negative mind-set, with the suggestion that compulsive self-starvation serves to reduce the anxiety and negative affect that becomes associated with food (Zink and Weinberger, 2010). Indeed, food ingestion may be experienced as anxiogenic for individuals with AN (Bailer et al., 2012). This may provide one mechanism through which individuals with AN experience self-starvation as a relief from this food associated anxiety, and indicates that negative reinforcement plays a big role in the maintenance of AN. Furthermore, compulsive exercise behavior has been associated with high levels of anxiety and depression in AN, and may serve as a means of reducing these negative states (Meyer et al., 2011).

However, the maintenance of restrictive behavior in AN may also involve positive reinforcement processes. Selby et al. (2014) suggest a model in which positive emotions associated with successful weight loss, such as feelings of pride and accomplishment, reinforce and drive further weight loss behavior. Over time, weight loss behavior is conditioned to elicit positive emotions regardless of whether further weight loss is attained (Selby et al., 2014). The underlying premise of this theory is consistent with that of Walsh’s habit formation theory (Walsh, 2013). Evidence that weight loss behavior in AN may be driven by positive reinforcement comes from a series of functional magnetic resonance imaging (fMRI) studies. Fladung et al. (2010) found that positive feelings and increased activation in the VS were associated with underweight pictures in individuals with AN, with reduced VS activation during the normal weight pictures. This was in comparison to healthy individuals, who showed the opposite pattern of activation in the VS. In a further study, Fladung et al. (2013) found that this VS signal may change over time, in the same way that neural activation evolves over time in substance dependence. In this second study, individuals with AN, whose illness duration was five times shorter than those in the first study, showed the same increased activation to underweight pictures and decreased activation to normal weight pictures in comparison to healthy controls. However, the signal did not differ between categories, suggesting that the signal in the VS may evolve over time, as the illness duration increases. The authors suggest that over time cues associated with self-starvation are linked to strong motivational value, which further reinforces starvation and weight loss behavior (Fladung et al., 2013). Increased positive feelings and a pronounced attentional bias to stimuli depicting physical activity has also been found in AN (Giel et al., 2013).

### A SHIFT FROM POSITIVE TO NEGATIVE REINFORCEMENT?

Selby et al. (2014) suggest that positive emotion/reinforcement may be particularly relevant in the initial stages of AN, with negative reinforcement becoming more important in maintaining behavior over time, where weight loss behavior is used to reduce negative emotions. It may be that the later stages of AN and drug dependence are similar, in that the early rewarding effects of food restriction and drug-taking may both cause these behaviors to be repeated and become habitual/compulsive in nature (Zink and Weinberger, 2010). This could be reflected by synaptic changes in both substance dependence and AN (Everitt and Robbins, 2005; Fladung et al., 2013).

The synaptic changes seen over time in drug dependence are well studied. The initial DA-dependent reinforcing effect of drugs is associated with a feeling of euphoria, and is experienced as rewarding (Volkow et al., 1996), thus positively reinforcing their use. These DA-dependent effects cause drugs themselves and drug-associated stimuli to gain incentive salience and promote further drug-seeking behavior (Everitt and Robbins, 2005). The repeated and prolonged DA increase results in synaptic changes in DA pathways, and these changes may be responsible for the formation of compulsive habits in substance dependence (Wolf, 2002). These later stages of habitual drug-seeking and taking may be negatively reinforced by the withdrawal symptoms experienced in absence of the drug, during which individuals can experience both physical and emotional negative states (Koob and Volkow, 2010). Protracted withdrawal is associated with hypofunctioning of the DA pathways and anhedonia (Volkow et al., 2007b), which may result in an increased drive for further drug-seeking to counteract this. Avoiding these withdrawal symptoms is likely to play a big role in reinforcing drug use, suggesting that the later stages of compulsive drug use may also involve negative reinforcement.

The complex interplay of positive and negative reinforcement in the development of compulsive behavior is also emphasized by habit formation studies in OCD. Whilst theories of habit formation in AN and substance dependence have been largely focused on appetitive habits resulting from initially rewarding outcomes, evidence suggests that OCD may be underpinned by the formation of avoidance habits. The compulsions seen in OCD do not seem to develop from an initially rewarding outcome, but rather are typically used to avoid some undesirable consequence (Gillan et al., 2013). These avoidant habits may be related to increased punishment sensitivity reported in OCD (Fullana et al., 2004), in which patients’ compulsions reduce distress, and as such may be experienced as an immediate avoidance of punishment (Salkovskis, 1999). If avoidance is important in the development of OCD, it may also have an impact on habit formation in AN and substance dependence. Individuals with AN have been shown to have both heightened reward and punishment sensitivity, suggested to reflect the avoidant and anxious behaviors seen in AN, which may be used as a means of avoiding increased emotional reactivity to reward and punishment (Jappe et al., 2011). As such, habits in AN may develop as an avoidance of the negative emotions associated with food and weight gain. However, substance dependence is associated with decreased reward and punishment sensitivity (de Ruiter et al., 2009). Whilst compulsive habit behaviors in OCD, appear to begin as the avoidance of something negative, initial drug taking in substance dependence may be much more dependent on increasing the experience of reward. This process is less clear in AN, in which behavior may begin as the pursuit of something rewarding (weight loss) and the avoidance of negative states and anxiety associated with food.

### SUMMARY

• Whilst AN is often associated with avoidance behaviors, it may in part involve the pursuit of the rewarding aspects of weight loss.
• Theories of substance dependence suggest a shift over time from positive to both positive and negative reinforcement, corresponding to a shift from goal directed to habitual behavior.
• Compulsive behavior in AN may reflect the later stages of substance dependence, in which compulsive habits are driven by both positive and negative reinforcement.
• Negative reinforcement appears to dominate the development of persistent compulsions in OCD.

## THE ROLE OF STARVATION IN COMPULSIVE BEHAVIOR

**FIGURE 3:**
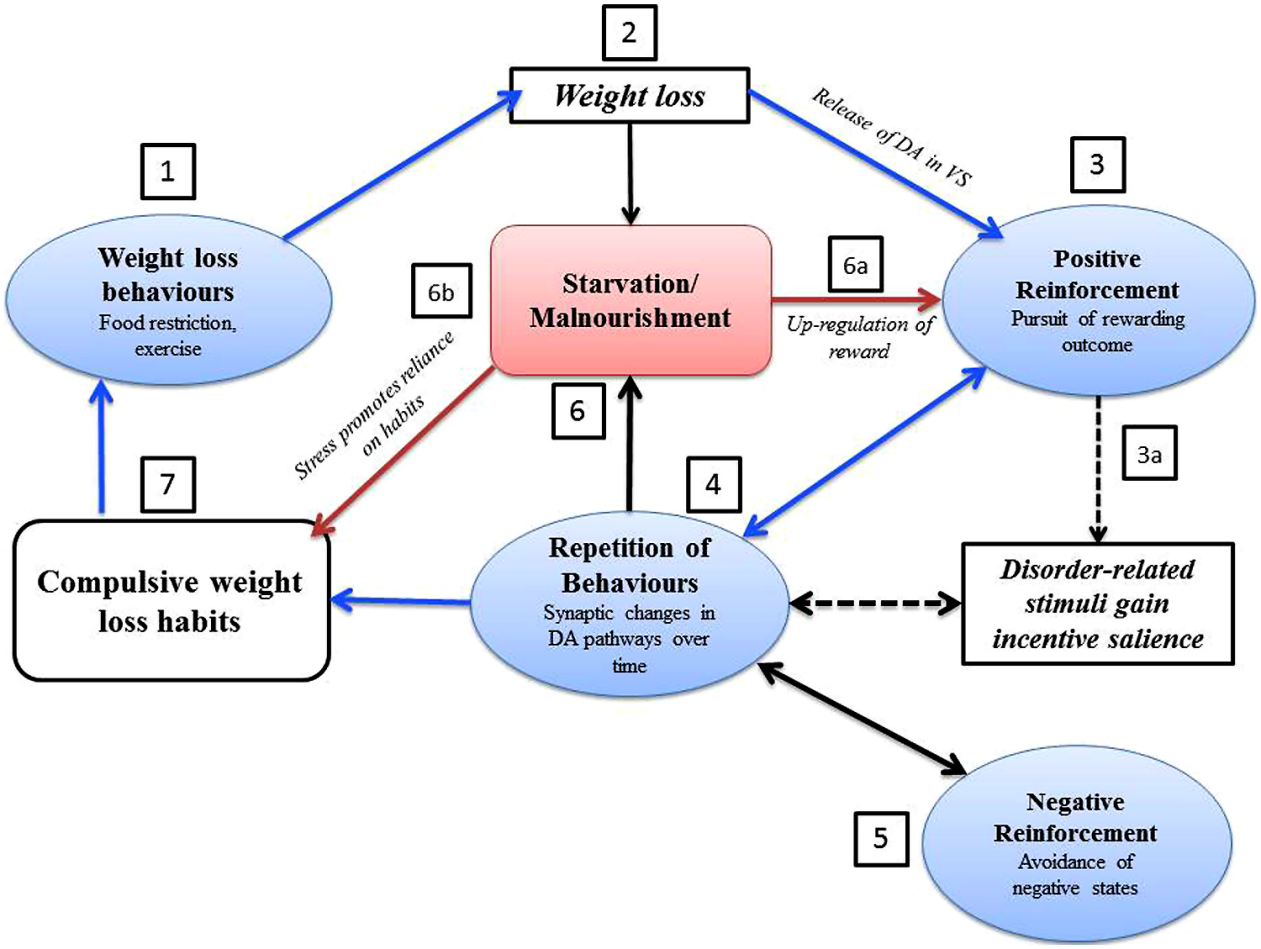
**The vicious circle of self-starvation in anorexia nervosa: representation of the development of compulsive weight loss habits in AN.** This can be conceptualized as a vicious circle, augmented by starvation. (1) Goal-directed weight loss behaviors, such as food restriction and exercise, lead to weight loss. (2) Weight loss is experienced as rewarding, which may be positively reinforced by an increase of DA in the VS. (3) The pursuit of the rewarding aspects of weight loss leads to a repetition of weight loss behaviors: (a) disorder related cues may gain incentive salience through conditioned reinforcement, and may themselves trigger weight loss behavior (Walsh, 2013). (4) Repetition of rewarding weight loss behavior may result in synaptic changes in DA pathways (Fladung et al., 2013) and result in DA hypofunctioning in a similar way to substance dependence (Everitt and Robbins, 2005). (5) The avoidance of negative states and the anxiety associated with food may become more important in reinforcing weight loss behaviors in the later stages of AN (Selby et al., 2014). (6) Starvation and malnourishment further promote weight loss behavior in the following ways: (a) the up regulation of reward associated with starvation may lead to disorder-related cues gaining incentive salience (Fladung et al., 2010) through a molecular cascade in which starvation induced reductions in glucose and insulin lead to an increase in phasic DA transmission in the VS (Zink and Weinberger, 2010). Furthermore, AN is associated with increased sensitivity to both reward and punishment (Jappe et al., 2011) which may enhance both positive and negative reinforcement of weight loss. (b) Stress is associated with a reliance on habits in learning (Schwabe et al., 2007), and the stress associated with starvation may promote the compulsive weight loss behaviors seen in AN. (7) Once weight loss behavior is repeated enough, a rewarding outcome may no longer be needed for behavior to continue, and weight loss behaviors may become compulsive and habitual (Walsh, 2013). Abbreviations: DA, dopamine; VS, ventral striatum.

The evidence reviewed so far suggests that compulsive weight loss behaviors seen in AN are associated with aberrant neurocircuitry and dysfunctional mechanisms of learning and reward that promote compulsive behavior. Crucially, there is evidence that the physiological consequences of starvation promote compulsivity and habit learning, and directly affect positive reinforcement (see **Figure 3** for a representation of the effects of starvation on the development of compulsive weight loss habits in AN). For example, in animal models DA (Verhagen et al., 2009b) and serotonin are released in the NAc during starvation-induced hyperactivity, while DA antagonism inhibits anorectic behavior (Verhagen et al., 2009a).

Behavioral evidence in humans suggesting that starvation is associated with the development of compulsive behaviors derives from the Minnesota Experiment (Keys et al., 1950), in which previously healthy males were restricted to half their average food intake for 6 months. These individuals developed food rituals and obsessions, some of which persisted after food restriction ceased, and some engaged in binge eating or excessive exercise. They also experienced cognitive impairment and periods of low mood during the study. These individuals were psychologically and physically healthy prior to the experiment, suggesting that these symptoms were due to the food restriction imposed during the experiment. It is possible that starvation effects impact on aberrant neurocircuitry, and dysfunctional mechanisms of learning and reward, to promote compulsive behavior in a vicious circle.

In the animal model of AN, activity based anorexia (ABA) compulsive hyperactivity becomes intrinsically linked to food restriction. Rats on a restricted diet given free access to a running wheel will increase activity to the point of death (Routtenberg and Kuznesof, 1967; Adan et al., 2011). Interestingly, young female rats, as in human AN, show particular susceptibility to this effect (Hancock and Grant, 2009). These rats also show food anticipation activity (FAA), in which an increase in physical activity precedes a meal (Mistlberger, 1994). This increase in activity preceding a meal has also been documented in AN patients (Scheurink et al., 2010). These phenomena have been suggested to reflect an evolutionary advantage of increased activity (foraging) during times of famine (Illius et al., 2002), and appear to show a link between food restriction and increased physical activity. Evidence from the ABA model also suggests a role for DA and serotonin dysfunction in the development of hyperactivity during food restriction. Antagonism of DA receptors is shown to increase food intake and decrease overall physical activity in ABA rats (Verhagen et al., 2009a). Similarly, DA depletion and DA receptor blockade in the NAc decreases FAA (McCullough and Salamone, 1992; Barbano and Cador, 2006). Serotonin has a suppressive effect on food intake (Simansky, 1996) and a decrease in food intake is seen in rats treated with serotonergic agonists (Clifton et al., 2000; Lee et al., 2002). Furthermore, a decrease in 5HT release in the NAc is seen in ABA rats (Verhagen et al., 2009b). Given that AN is associated with altered DA and serotonin levels (see Neurotransmitter Involvement in Compulsivity), this evidence suggests that alterations in these neurotransmitters during the chronic food restriction of AN may increase vulnerability to compulsive exercising.

Food restriction has also been linked to an increase in reward sensitivity. Chronic food restriction, resulting in significant body weight loss, has been shown to increase reward effectiveness when electrically stimulating brain reward circuitry in rats (Fulton et al., 2004). Moreover, body weight correlates with the stimulation threshold for reward, in that a lower body weight leads to a weaker stimulation threshold (Abrahamsen et al., 1995). This effect can also be seen in drug reward. Indeed, chronic food restriction in rats has been found to enhance the rewarding properties of drugs by up-regulating synaptic plasticity in the NAc (Carr, 2007). This increased sensitivity to reward during food restriction may underpin reports of increased salience and attention for disorder-related cues in AN (Fladung et al., 2010, 2013; Giel et al., 2013). Zink and Weinberger (2010) suggest that these disorder-related cues gain incentive salience through a molecular cascade in which starvation induced reductions in glucose and insulin lead to an increase in phasic DA transmission in the VS, a process which conditions associated cues to become highly motivationally salient. This salience increases the likelihood that the behavior will continue, in the same way as drug-associated cues in substance dependence (Zink and Weinberger, 2010). In this way, the neurobiological changes associated with chronic food restriction may enhance the experience of reward in AN, and positively reinforce disorder related compulsions.

The reliance on habits or stimulus-response learning suggested to lead to compulsive behavior may be potentiated in times of stress. Stress is shown to modulate cognitive memory systems in favor of neo-striatum dependent habit systems (Schwabe et al., 2007). Furthermore, participants exposed to an experimental stressor have been shown subsequently to rely on habits during an instrumental learning task, and to show reduced knowledge of the action-outcome associations needed for goal-directed behavior (Schwabe and Wolf, 2009). Food shortage has also been associated with impairments in memory in animals (Plaçais and Preat, 2013), and even brief food restriction is shown to lead to alterations in gene expression of stress hormones (Guarnieri et al., 2012). Thus in AN, psychological and physical stressors associated with the disorder, as well as chronic food restriction, may promote reliance on habits.

### SUMMARY

• Starvation and weight loss is associated with physiological changes that promote compulsive behavior.
• The ABA animal model of AN links food restriction to hyperactivity, and alterations in DA and serotonin are seen in ABA rats.
• Food restriction is associated with an increase in reward sensitivity and positive reinforcement.
• An increase in stress prior to learning is associated with dependence on stimulus-response habits in humans.

## IMPLICATIONS FOR TREATMENT

The behavioral and neurobiological research reviewed suggests that compulsivity is a construct that can be seen in varying degrees across disorders such as AN, OCD, and substance dependence. Conceptualizing compulsive behavior across diagnostic categories opens the way for transdiagnostic treatment strategies. These may target common processes underpinning the compulsivity seen in disorders such as AN, OCD, and substance dependence. The dysregulation of habit formation and reward processes suggested to underpin the development of compulsivity is a potential target for these novel treatment strategies. Treatments targeted at disrupting habitual behavior may have transdiagnostic efficacy.

Functional magnetic resonance imaging findings suggest that the neural areas associated with habit learning and goal directed behavior may be dysfunctional during symptom provocation in AN, providing a potential neural underpinning for an overreliance on habit formation at the expense of goal directed actions. A model of habit formation in AN may thus provide a testable explanation of the development of the persistent compulsive behaviors that develop during the disorder.

Re-patterning habitual behaviors, alongside reversing starvation, has been emphasized as an important component in the treatment of AN (Park et al., 2011, 2012); some concepts previously aimed at behavioral change and habit breaking in other disorders have been translated for use in AN. Given the effects of starvation on neurobiological maintaining mechanisms reviewed above, it is likely to be of crucial importance to reverse starvation effects in tandem. Examples of habit breaking strategies are components of cognitive remediation therapy (CRT) and exposure response therapy (ERT). CRT was originally developed for use in the treatment of psychosis, and has also been adapted for use in eating disorders to improve cognitive flexibility and break cognitive habits. Preliminary research into CRT has found improvements in self-reported cognitive flexibility in individuals with AN (Tchanturia et al., 2007a). ERT, which targets conditioned fear responses and conditioned reward, has been used in the treatment of OCD (Foa et al., 2005) and addiction (Kaplan et al., 2011), and some success has been found with graded exposure to food cues in AN, reducing meal-related anxiety post-treatment (Steinglass et al., 2012a).

Given evidence of neural circuitry underpinning compulsivity across disorders, neuromodulatory interventions such as deep brain stimulation (DBS), and the less invasive repetitive transcranial magnetic stimulation (rTMS), may benefit from common neural targets for treatment across disorders. DBS is a reversible, adjustable neurosurgical treatment that involves implanting electrodes that send electrical impulses to chosen locations in the brain (Rauch, 2003). DBS has been effective in the treatment of OCD when targeting the NAc, and is suggested to normalize excessive connectivity between the NAc and PFC (Figee et al., 2013). The reported efficacy of DBS to the NAc in OCD and addictions (Kuhn et al., 2007; Liu et al., 2008; Muller et al., 2009; Denys et al., 2010; Figee et al., 2013) is consistent with the involvement of fronto-striatal circuits across compulsive disorders. Both animal studies (Liu et al., 2008; Henderson et al., 2010) and DBS in substance dependent individuals (Kuhn et al., 2007; Muller et al., 2009) have found decreases in addictive behaviors when stimulating the NAc. Given that the NAc area is suggested to be involved in the transition from voluntary to compulsive drug use (Everitt and Robbins, 2005), it is possible that NAc stimulation may disrupt this process and thereby reduce compulsive behaviors. Case reports of DBS to the NAc in AN patients have also reported symptom alleviation both in the presence and absence of comorbid OCD (Wu et al., 2012; Lipsman et al., 2013; McLaughlin et al., 2013). RTMS, a non-invasive brain stimulation technique targeting the dorsolateral prefrontal cortex, has been shown to have some efficacy across disorders, reducing cravings and consumption in substance dependence (Barr et al., 2011), decreasing compulsions and obsessions in OCD (Blom et al., 2011), and reducing anxiety and potentially the urge to exercise in AN (Van den Eynde et al., 2013).

Novel pharmacological treatments targeting addictive or compulsive behaviors may also prove useful in the treatment of compulsivity across disorders. The DA dysfunction seen in many compulsive disorders may be a potential target for pharmacological intervention. Given animal and human evidence that DA modulation of glutaminergic transmission plays a role in reward and reinforcement, glutaminergic medications may also prove useful in compulsive disorders such as addictions (Kenny and Markou, 2004). The use of drugs that target GABA and glutamate pathways has shown some benefit in the treatment of substance use disorders (Clarke et al., 2004; Olive et al., 2012), binge eating (Guardia et al., 2011), and OCD (Pittenger et al., 2011), and may also prove beneficial in other disorders in which compulsive behavior develops.

## CONCLUDING REMARKS

Conceptualizing compulsivity as a transdiagnostic concept, uniting separately classified disorders through common pathological processes, may help in the development of novel behavioral and neural interventions, which could be effective across diagnostic boundaries. These are urgently needed, given the poor outcome and limited evidence base for treatment of AN, especially in adulthood. Future research should aim to test this concept directly, for example by looking at the behavioral and neural basis of habit formation in relation to compulsivity in AN, OCD, and substance dependence, as well as other disorders that exhibit compulsive behaviors.

## Conflict of Interest Statement

The authors declare that the research was conducted in the absence of any commercial or financial relationships that could be construed as a potential conflict of interest.
